# Turning to the negative: attention allocation to emotional faces in adolescents with dysregulation profile—an event-related potential study

**DOI:** 10.1007/s00702-021-02319-x

**Published:** 2021-03-10

**Authors:** Franziska Martin, Marlies Pinnow, Stephan Getzmann, Stefan Hans, Martin Holtmann, Tanja Legenbauer

**Affiliations:** 1 LWL-University Hospital for Child and Adolescent Psychiatry, Ruhr-University Bochum, Hamm, Germany; 2 Department Biopsychology, Marlies Pinnow, Ruhr-University Bochum, Bochum, Germany; 3 Leibniz Research Centre for Working Environment and Human Factors at the Technical University of Dortmund (IfADo), Dortmund, Germany

**Keywords:** Dysregulation profile, Attentional network, Face processing, Event-related potentials

## Abstract

Patients with irritability, temper outbursts, hyperactivity and mood swings often meet the dysregulation profile (DP) of the Child Behavior Checklist (CBCL) or the Strengths and Difficulties Questionnaire (SDQ), which have been investigated over the past few decades. While the DP has emerged as a transdiagnostic marker with a negative impact on therapeutic outcome and psychosocial functioning, little is known about its underlying mechanisms such as attention and emotion regulation processes. In this study, we tested whether adolescent psychiatric patients (*n* = 27) with the SDQ-DP show impaired emotional face processing for task-irrelevant stimuli compared to psychiatric patients without the SDQ-DP (*n* = 30) and non-clinical adolescents (*n* = 21). Facial processing was tested with event-related potential (ERP) measures known to be modulated by attention (i.e., P1, N1, N170, P2, and Nc) during a modified Attention Network Task, to which task-irrelevant emotional stimuli (sad, fearful, and neutral faces) were added prior to the actual trial. The results reveal group differences in the orienting and in the conflicting network. Patients with DP showed a less efficient orienting network and the clinical control group showed a less efficient conflicting network. Moreover, patients with the dysregulation profile had a shorter N1/N170 latency than did the two control groups, suggesting that dysregulation in adolescents is associated with a faster but less arousing encoding of (task-irrelevant) emotional information and less top-down control.

## Introduction

A large proportion of children and adolescents who are referred to clinical treatment present with self-regulation difficulties in emotional, behavioral and cognitive domains (Deutz et al. 2018; Holtmann et al. 2007). These are not unique to a specific diagnostic category (Ayer et al. 2009; Carballo et al. 2014; Legenbauer et al. 2018), but are rather described within the transdiagnostic so-called dysregulation profile (DP; Deutz et al. 2018, 2020; Althoff et al. 2010; Ayer et al. 2009). The DP reflects a profile of elevated scores on subscales of the widely used Child Behavior Checklist (CBCL; Achenbach 1991) or the Strengths and Difficulties Questionnaire (SDQ; Goodman et al. 1998; Holtmann et al. 2011; Deutz et al. 2018; Wang et al. 2018).

There is extensive research validating the clinical utility of the DP (Carballo et al. 2014) with respect to its presence in the case of psychosocial impairments (Holtmann et al. 2008; Jucksch et al. 2011; Wang et al. 2018), worse family functioning (Biederman et al. 2009; Carballo et al. 2014), specific genetic profiles (Hudziak et al. 2005) and biological correlates (e.g., Holtmann et al. 2010b). Given that the DP is stable over time and across age groups (Carballo et al. 2014; Wang et al. 2019) and has been related to higher suicide rates, more substance use and higher rates of depression and anxiety disorders when emerging in adulthood (Holtmann et al. 2010a; Metzke and Steinhausen 2019), it is important to better understand what lies beneath these specific self-regulation difficulties. To achieve this, the inspection of attentional and emotion regulating processes may be a promising candidate, because self-regulation difficulties have been associated with impaired attention allocation to emotional stimuli in stressful situations in children with emotional dysregulation.

The attention system is linked to the emotion system via a reciprocal relationship (e.g., Pourtois et al. 2013). Whereas emotional stimuli have a modulatory (usually capturing) effect on attention (for review, Carretié 2014), findings from neurobiological studies showed that the processing of emotional stimuli also depends on the extent to which attentional resources are available (Morawetz et al. 2010; see also Yamaguchi and Onada 2012). Consequently, disturbances in attentional processes may underlie self-regulation difficulties. Self-regulation difficulties have been associated with impaired attention allocation in motivational and frustrating situations. Those self-regulation difficulties were found to be accompanied by a reduced amplitude of the N100 event-related potential (ERP; Rich et al. 2007), suggesting impairments in the initial stages of attention.

Electrophysiological methods, like the analysis of ERPs to emotional faces may help to elucidate the allocation of attention and the processing of emotional stimuli (e.g., Eimer and Holmes 2007; Dennis et al. 2009). In particular, negative emotional stimuli, like fearful or sad faces, have been shown to elicit higher amplitudes in early ERPs, in ERPs reflecting the processing of emotional salience, and in ERPs reflecting attentional processes. The first ERPs to be mentioned here are the P1 und N1, a positivity over occipital scalp areas and a negativity over centro-parietal areas, which peak about 100 ms after stimulus onset. P1 and N1 are traditionally related to sensory gain control and the early processes of stimulus detection and selective attention, respectively (Mangun 1995), and both are sensitive to the emotional expression of faces (e.g., Batty and Taylor 2003; Eimer and Holmes 2002; Dennis et al. 2009). The next ERP of interest is the N170, a posterior-occipital negativity peaking around 170 ms after stimulus onset, which is assumed to reflect an early stage of face encoding (e.g., Batty and Taylor 2003; Bentin et al. 1996; Leppänen et al. 2007; Righart and de Gelder 2008). Although there are some contradictory findings (see e.g., Eimer et al. 2007), there is some evidence that the N170 is modulated by facial expressions of emotion, being increased in amplitude especially by fearful expressions (Batty and Taylor 2003; Leppänen et al. 2007; Righart and de Gelder 2008). The N170 is followed by the P2, which is also associated with stimulus processing in general and is modulated by attention, likely reflecting a filter mechanism involved in the allocation of attention (Lijffijt et al. 2009). The P2 amplitude is higher for emotionally salient stimuli (e.g., negative emotional faces; Carretié et al. 2001). A final ERP associated with face processing in children and adolescents is the negative central component (Nc). This ERP occurs around 500 ms after stimulus onset at fronto-central electrodes and is linked to attentional and memory processes (Richards et al. 2010). It was shown that Nc amplitude is larger during sustained attention than during inattention (Richards et al. 2010) and after negative versus positive emotional faces (Nelson and De Haan 1996). Findings like these suggest an early disturbance of attentional deployment when emotional stimuli are involved.

Additional evidence from cognitive and neuroscientific research using the Attention Network Task (ANT; Fan et al. 2002) emphasizes the reciprocal relationship between emotion and attention. Originally, the ANT was applied to precisely capture attention deficits, and to allocate the differential deficit across three attentional networks, namely alerting (allowing a state of alertness to be achieved and maintained), orienting (allowing information from sensory input to be selected by directing or disengaging attention to one stimulus among others and/or shifting the attentional resources from one stimulus to another), and executive control (involving the top-down control of attention and allowing response conflicts to be resolved) (Fan et al. 2002; Heeren et al. 2015). The ANT is applicable for adults as well as for children and adolescents. Although most research on the ANT has been done with adult participants, it has been shown that attention network scores and errors do not differ between adults and children (Rueda et al. 2004). Modified versions incorporating emotional distractors were developed (e.g., Dennis et al. 2009; Cohen et al. 2011), which display task-irrelevant emotional stimuli before the actual tasks (e.g., sad, fearful, or neutral faces or pictures with negative emotional salience from the International Affective Picture System (IAPS); e.g., Cohen et al. 2011; Dennis et al. 2008). These studies found an impact of emotion on attention. For example, anxiety-relevant information (e.g., the fearful faces) led to decreased efficiency of the alerting network (Cohen et al. 2011). Additionally, a study by Heeren et al. (2015) found that in patients with social anxiety disorder (SAD), the orienting network was impaired compared to healthy controls, which was attributed to faster attentional engagement to the task-irrelevant information.

To date, there are no studies in children and adolescents who meet the DP, despite the evidence relating attention and emotion in this group. Consequently, the present study seeks to explore attention allocation in children and adolescents with DP. We expected to see less efficient attention networks in the DP group, and especially the alerting network should show deficits relative to the control groups. In order to reveal potential neurocognitive mechanisms associated with these specific deficits, emotion-induced modulations of ERPs were analyzed and contrasted in the DP and control groups. Specifically, modulations in P1 and N1/N170 (i.e., reduced amplitudes in the DP group) should reveal deficits in sensory gain control, early processes of stimulus detection, selective attention, and face encoding, while those in P2 and Nc would hint at deficits in later processes of allocation of attention and memory processes. In addition to ERP amplitudes, latencies were analyzed in order to detect potential differences between DP and control groups in the temporal processing of emotional stimuli.

## Methods

### Participants

In total, 78 participants (56 females) aged between 12 and 17 years were recruited as part of a larger study at the LWL-University Hospital for Child and Adolescent Psychiatry in Hamm, Germany, and at a local high school. Other data from the larger study are published elsewhere (Legenbauer et al. 2018). Participants were assigned to three groups, which were based on the SDQ-dysregulation profile scores (SDQ-DP; Goodman et al. 1998; Holtmann et al. 2011; Deutz et al. 2018) and were defined according to Holtmann et al. (2011) as follows: Patients scoring 5 or higher on the SDQ-DP were assigned to the clinical group with DP (DPG), whereas patients scoring below this cutoff were assigned to the clinical control group (CCG). Participants recruited at the high school were also screened for the SDQ-DP. If they did not show the SDQ-DP, they were included as part of the non-clinical control group (NCG); otherwise, they were excluded.

General exclusion criteria were an IQ below 85, neurological disorders, post-traumatic stress disorder, autism, drug abuse, pregnancy, acute intoxication, or benzodiazepine intake. Participants of the NCG were also excluded if they reported a history of psychiatric disorders. All participants and their legal guardians provided informed consent.

### Assessments

Participants, whose data are presented here, also performed two other experiments on the relationship between dysregulation profile and attention and dysregulation profile and emotion recognition (for more information see Legenbauer et al. 2018). Table 1 provides descriptive data about the participants in each group.

**Table 1:** Demographic data of the present sample divided into groups based on SDQ-DP score

	DPG	CCG	NCG
Sample size	27	30	21
Number female	22 (81%)	16 (53.3%)	18 (85.7%)
Age in years	14.70 (1.49)	14.67 (1.56)	13.67 (1.46)
SDQ			
Emotional Problems	6.59 (2.17)	4.53 (2.90)	2.86 (1.88)
Conduct Problems	3.93 (2.77)	2.33 (2.02)	1.19 (0.93)
Hyperactivity	5.81 (1.76)	3.90 (2.60)	2.86 (2.15)
Peer Interaction	5.48 (2.42)	3.13 (2.08)	1.57 (1.60)
Prosocial Behavior	6.93 (2.95)	6.97 (2.79)	8.62 (1.47)
Dysregulation Profile	5.74 (1.10)	2.97 (1.03)	1.81 (1.94)
Diagnoses			
Depressive disorders	14 (51.9%)	12 (40%)	–
Anxiety disorders	7 (25.9%)	9 (30%)	–
Hyperkinetic disorders	6 (22.2%)	4 (13.3%)	–
Disorders of conduct and emotions	5 (18.5%)	7 (23.3%)	–
Other diagnoses	6 (22.2%)	10 (33.3%)	–
Medication			
Stimulants	2 (7.4%)	3 (10.0%)	–
SSRIs	9 (33.3%)	5 (16.6%)	–
SSNRIs	–	1 (3.3%)	–
Neuroleptics	2 (7.4%)	2 (6.7%)	–
Hormones	2 (7.4%)	1 (3.3%)	–
IQ	99.75 (11.73)	103.55 (11.88)	110.80 (13.94)

*DP*G clinical patients with DP, *CCG* clinical control group, *NCG* non-clinical control group, *SSRIs* selective serotonin reuptake inhibitors, *SSNRIs* selective serotonin-norepinephrine reuptake inhibitors

### Strengths and difficulties questionnaire

Participants answered demographic questions and completed the “Strengths and Difficulties Questionnaire” (SDQ; Goodman et al. 1998; German normation: Becker et al. 2018) to determine group assignment. The SDQ is a self-report questionnaire containing 25 statements about emotional problems, conduct problems, hyperactivity, peer interaction and prosocial behavior. The statements are rated on a 3-point Likert scale from “not true” to “certainly true”. The SDQ showed good internal consistency in the present sample, with Cronbach’s α = 0.76. The SDQ-DP represents the sum of five SDQ items, encompassing two questions regarding emotional problems, two questions regarding conduct problems and one question regarding hyperactivity.

### Attention network task

The attention network task (ANT) is a combination of a cued reaction time task and an Eriksen flanker task (Eriksen and Eriksen 1974; Fan et al. 2002). It is capable of measuring the efficacy of three attentional networks by scoring and combining the reaction times of different cue and flanker conditions. In the present study, the ANT was modulated and preceded by emotional faces that displayed either sadness, fear, or a neutral expression (Dennis and Chen 2007) but did not provide any other task-relevant information. The faces were taken from the NimStim stimulus set (Tottenham et al. 2009). After a face was shown, a cue appeared, followed by a target arrow, which randomly occurred above or below a fixation cross. The target arrow was surrounded by four flanker arrows, two on each side. The participant’s task was to indicate, by pressing the left mouse button with the left thumb or the right mouse button with the right thumb, whether the central arrow points to the left or to the right. Reaction time (RT) was measured as the time between the onset of the central arrow and the button press. During the task, participants were seated 60 cm from a 22-inch screen, which presented the stimuli with a resolution of 1680 × 1050 pixels. The task was programmed in E-Prime, Psychology Software Tools, version 2.

**Fig. 1:**
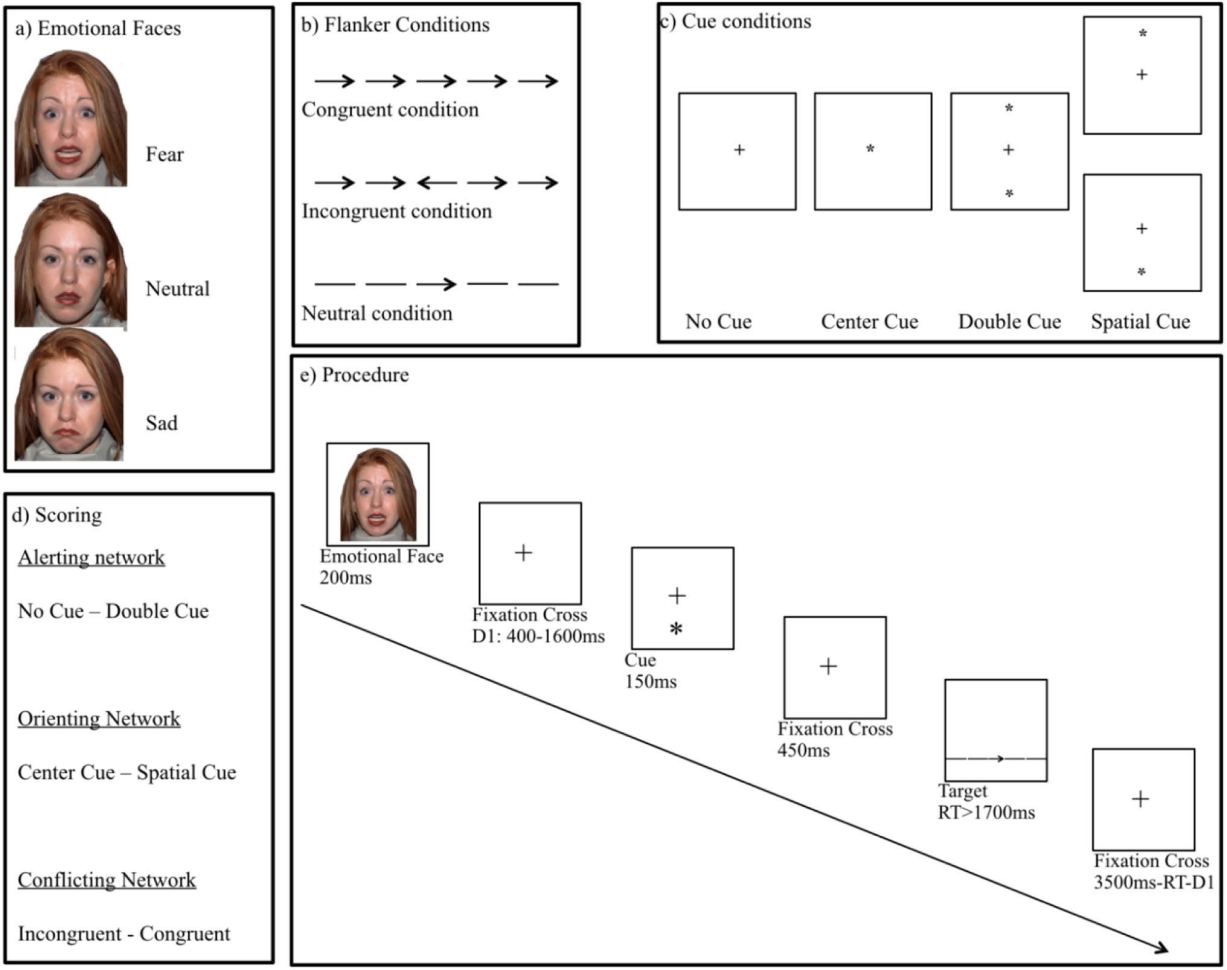
Summary of **a** the emotional cues, **b** the flanker conditions, **c** the cue conditions, **d** the scoring of the attention networks, and **e** the temporal procedure of the Attention Network Task. In this example, a sad face precedes a spatial cue neutral trial (based on Fan et al. 2002)

Figure 1 shows one trial of the ANT and the different cue and flanker conditions. There was a “no-cue” condition without warning cues, a “double-cue” condition, in which one cue appeared above and one below the fixation cross, a “center-cue” condition, in which the cue appeared superimposed over the fixation cross, and a “spatial-cue” condition, in which the upcoming target position was precisely indicated by the cue position.

Additionally, there were three flanker conditions: a congruent condition, in which the target and the flankers pointed in the same direction, an incongruent condition, in which the target and the flankers pointed in opposite directions, and a neutral condition, in which the flankers did not contain any spatial information.

After a training session of 24 trials, the actual experiment consisting of two blocks with 72 trials each was started. One block contained sad and neutral faces and the other block contained fearful and neutral faces. The order of the blocks was randomized per participant. Within each block, the emotional faces were pseudo-randomly presented. The faces were presented for 200 ms, followed by the fixation cross for 400–1600 ms. Next, the cue appeared for 150 ms, before a fixation cross was again presented for 450 ms. The target and the flankers appeared simultaneously until the participant reacted, not exceeding 1700 ms before a new fixation cross.

The efficiency of each attentional network was calculated using the RTs in different cue conditions and flanker conditions (Fan et al. 2002). The efficiency of the alerting network was determined as the RT difference between the no-cue minus double-cue condition, and the efficiency of the orienting network was calculated as the RT difference between the center-cue minus spatial-cue condition. The efficiency of the conflicting network was defined as the RT difference between the incongruent minus congruent condition. In all cases, higher scores indicate less efficient networks (Dennis and Chen 2007).

### EEG data recording

EEG was recorded from 32 Ag–Cl electrodes using the Brain Vision Recorder (Brain Products, Gilching, Germany). The continuous EEG was sampled at 250 Hz using the BrainAmp DC amplifier. Electrodes were applied according to the 10–20 system (Jasper 1924). Head size was measured and the position of the central electrode (Cz) was determined as the midpoint of the distance from nasion to inion and the distance between the ears. Vertical eye positions were recorded by electrooculography (EOG) using two electrodes positioned above and below the right eye. Electrode impedance was kept below 10 kΩ. Participants were asked to relax and to move as little as possible during the experiment.

### Analyses

#### EEG data processing

Raw data were visually screened for artifacts, and broken channels were restored by topographical interpolation. The data were re-referenced to the average of the 30 EEG channels and digitally band-pass filtered (cut-off frequencies 0.5 and 25 Hz). For further analyses, segments from 200 ms before to 1000 ms after the onset of each face stimulus were extracted. Data exceeding a maximum–minimum difference of 200 µV, a maximum voltage step of 50 µV per sampling point, or an activity of less than 0.5 μV within a 100-ms interval, were excluded from further analysis (automatic artifact rejection as implemented in the BrainVision Analyzer software, Version 2.0; Brain Products, Gilching, Germany). The EEG channels were corrected for ocular artifacts using the BrainVision Analyzer Independent Component Analysis tool. Again, an automatic artifact correction was performed (see above) and the remaining epochs were baseline-corrected to a 200-ms prestimulus window relative to the face stimulus onsets. Finally, the epochs were averaged, separately for neutral, fearful and sad emotional faces. For the analysis of the event-related potentials (ERPs), the mean peak amplitudes of P1, N1, N170, Nc and P2 were defined as local maximum positivity or negativity within time windows of the specific waveforms and across clusters of electrodes in which the ERPs were most pronounced (P1: 70–150 ms across occipital electrodes O1, Oz, O2; N1: 80–160 ms across centro-parietal electrodes CP1 and CP2; N170: 120–200 ms across parieto-occipital electrodes P7, P8, PO9, PO10; P2: 200–370 ms across parieto-occipital electrodes P7, P8, PO9, PO10; Nc: 290–370 ms across central electrodes C3, Cz, C4; each relative to the onset of the emotional faces). The choice of the time windows and electrode clusters was based on previous findings on the characteristics of the ERPs to emotional faces (e.g., Dennis and Chen 2007; Dennis et al. 2009) and confirmed by visual inspection of the grand average waveforms.

### Statistical analyses

All statistical analyses were performed using IBM^®^ SPSS^®^ Statistics version 24. Group differences for demographic data were calculated using one-way ANOVA. ERP mean amplitudes and latencies were submitted to repeated measures 3 × 3 ANOVAs with emotion (sad, fearful, neutral) as within-subject factor and group (DPG, CCG, NCG) as between-subject factor. Post-hoc comparisons are based on pairwise Bonferroni–Holm-corrected t-tests. For the attentional network RT data a 3 × 3 × 3 ANOVA was performed with network type (executive, orienting, alerting) and emotion (sad, fearful, neutral) as within-subject factors, and group (DPG, CCG, NCG) as between-subject factor. Post-hoc one-way ANOVAs were calculated to unravel significant main or interaction effects. For all results, the significance level was set at *α* < 0.05.

## Results

### Descriptive statistics

One-way ANOVAs revealed main effects of age [*F*(2,75) = 3.49; *p* < 0.05] and IQ [*F*(2,55) = 3.22; *p* < 0.05], but post-hoc t-tests did not reveal significant group differences (all *p*s > 0.05; Bonferroni-corrected). However, the groups differed with regard to BDI sum scores [*F*(2,74) = 7.07; *p* < 0.005], with DPG showing higher scores than CCG (*p* < 0.01) and NCG (*p* < 0.01). The two clinical groups did not differ regarding the sum of diagnoses (*p* = 0.50).

**Fig. 2:**
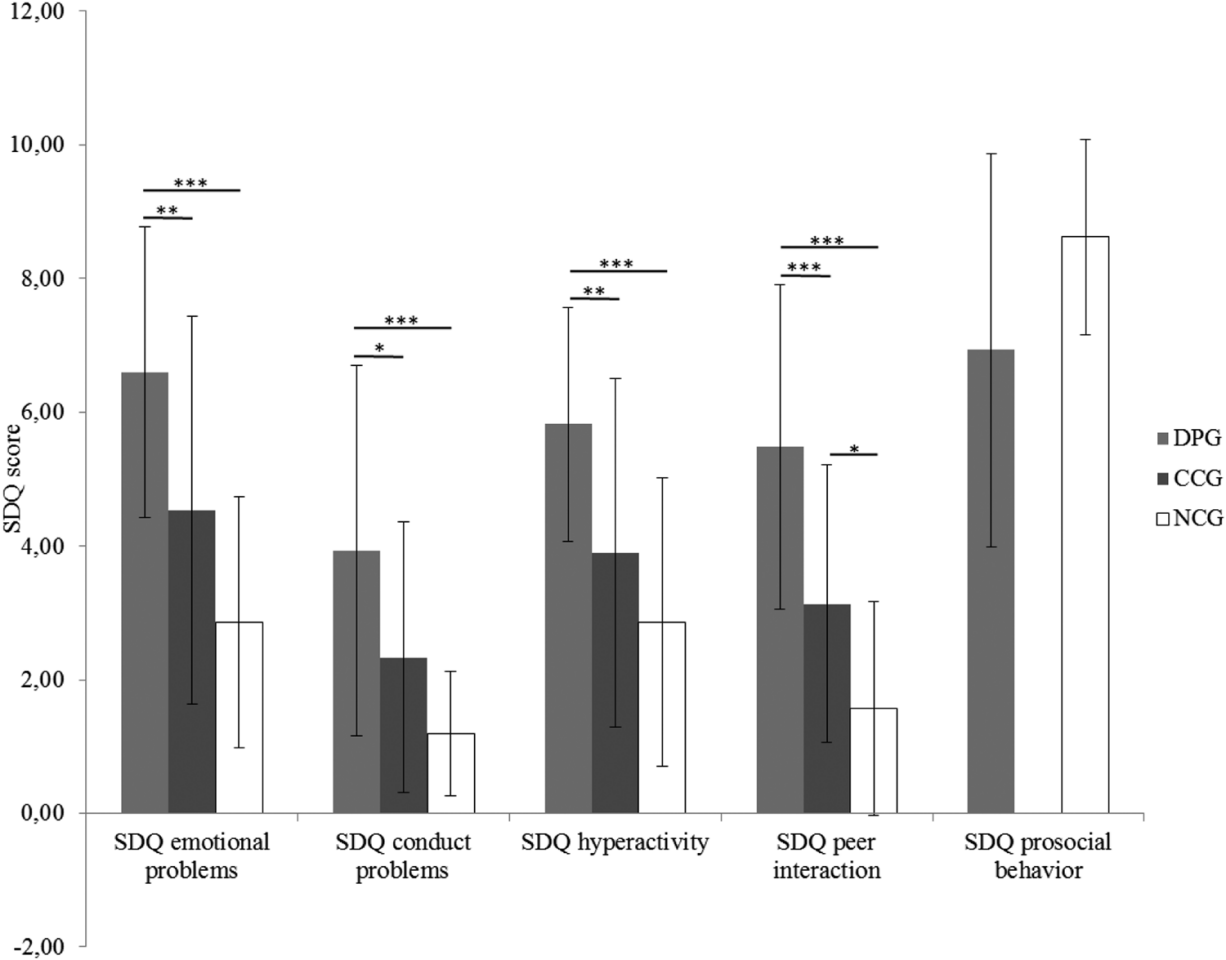
Group differences in SDQ scales (*DPG* clinical group with DP, *CCG* clinical control group without DP, *NCG* non-clinical control group, *SDQ* strengths and difficulties questionnaire)

The groups differed significantly regarding the SDQ scales “emotional problems”, “conduct problems”, “hyperactivity”, “peer interaction” and also regarding “prosocial behavior” (all *p*s < 0.044). Bonferroni-corrected post-hoc t-tests indicated that the DPG differed significantly from the CCG with respect to emotional problems and hyperactivity (both *p*s < 0.01), conduct problems (*p* < 0.05) and peer interaction (*p* < 0.001). Patients with DP also differed significantly from the NCG group on the scales emotional problems, conduct problems, hyperactivity and peer interaction (all *p*s < 0.001). Patients in the DPG showed the highest scores on all scales. Additionally, the CCG differed significantly from the NCG group on the peer interaction scale (*p* < 0.05). A post-hoc t-test was unable to resolve the group differences regarding prosocial behavior (both *p*s > 0.08). In sum, the analyses of the questionnaire data indicated that patients with DP show the strongest impairments in emotional problems, hyperactivity, conduct problems and peer interaction as measured with the SDQ. The CCG reported more problems in peer interaction than did the NCG (Fig. 2).

### Behavioral ANT data

**Fig. 3:**
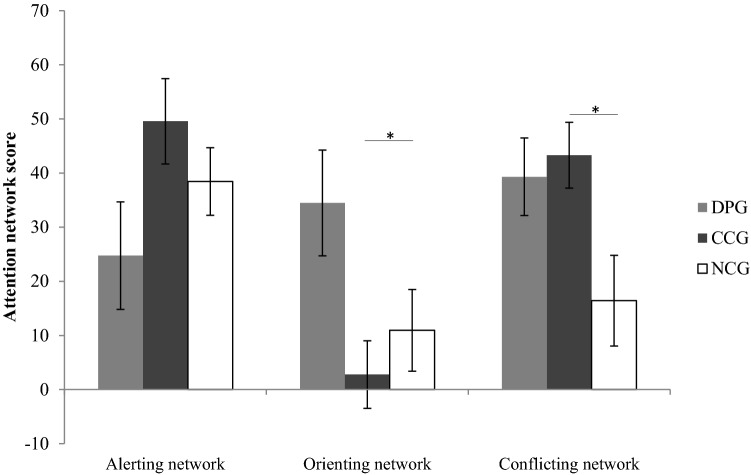
Values of the attention networks for each group. The higher the values, the less efficiently the network functions (*DPG* clinical group with DP, *CCG* clinical control group without DP, *NCG* non-clinical control group)

There was a main effect of attentional network [*F*(2,150) = 6.62,* p* < 0.01; *η*_p_^2^ = 0.081]. Post-hoc pairwise t-tests showed that the orienting network differed significantly from the alerting (*p* = 0.023) and the conflicting network (*p* = 0.002). More importantly, there was an interaction of network × group [*F*(4,150) = 4.82,* p* < 0.01; *η*_p_^2^ = 0.114]. To clarify this interaction, one-way ANOVAs were conducted, revealing that the groups differed in the orienting network [*F*(2,77) = 4.51, *p* < 0.05] and in the conflicting network [*F*(2,77) = 3.76, *p* < 0.05], but not in the alerting network (*p* = 0.10). The DPG showed significantly higher values than the CCG in the orienting network (*p* < 0.05). In the conflicting network, the NCG showed significantly lower values than the CCG (*p* < 0.05). Figure 3 shows the group differences in the attention networks.

### ERP data

**Fig. 4:**
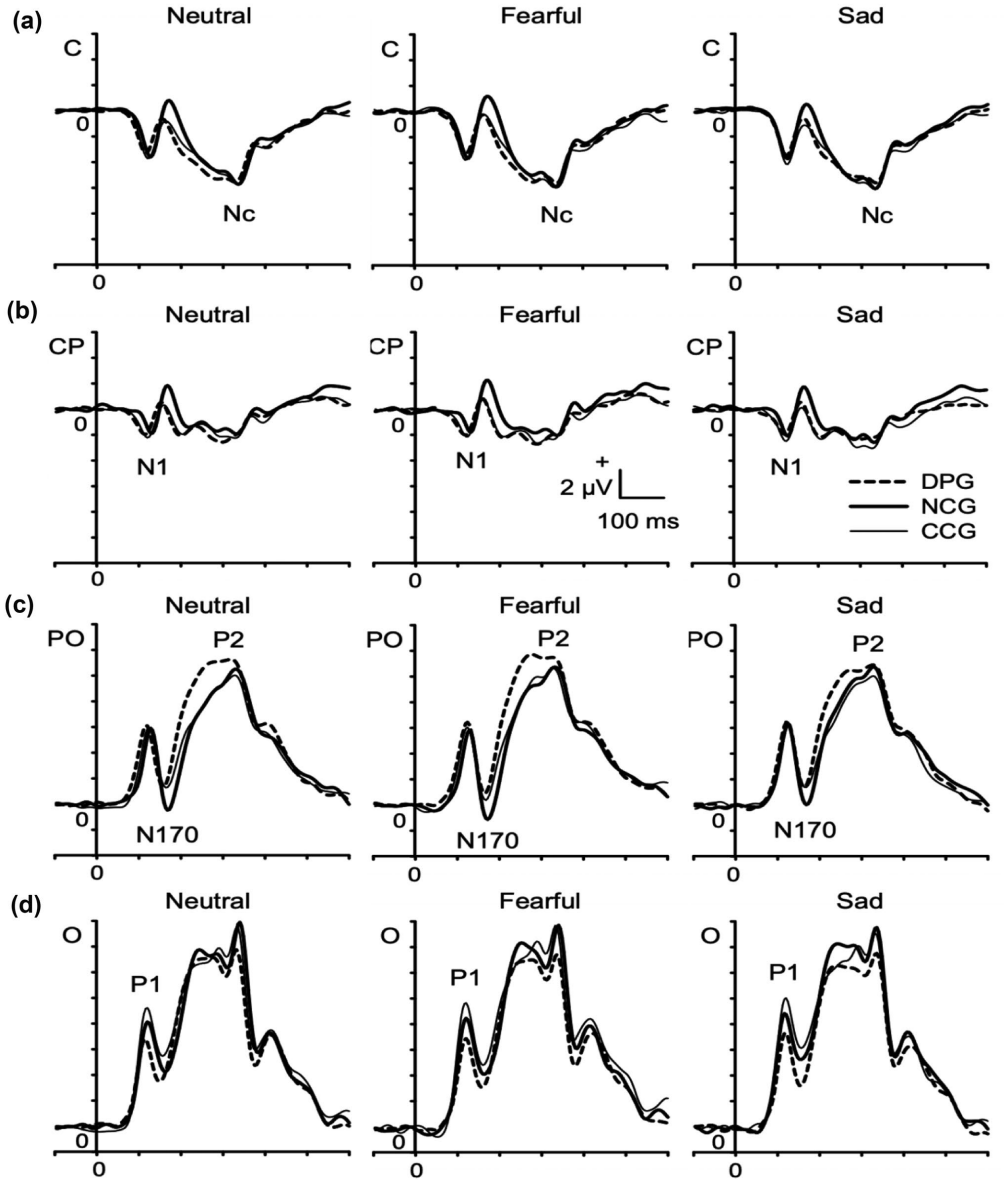
Grand average waveforms of the three groups (*DPG* clinical group with DP, *CCG* clinical control group without DP, *NCG* non-clinical control group), stimulus-locked to the onset of the emotional faces and averaged across arrays of **a** central C, **b** centro-parietal CP, **c** parieto-occipital PO, and **d** occipital O electrodes. Waveforms are shown separately for neutral, fearful, and sad faces, ERPs are marked

P1. There were no main effects of group or emotion on P1 mean amplitude or latency, and no interactions (all *F*s < 2.10; all *p*s > 0.12; all *η*_p_^2^ < 0.03; Fig. 4d).

N1. There were no main effects or interactions on N1 amplitude (all *F*s < 1.16; all *p*s > 0.33; all *η*_p_^2^ < 0.03; Fig. 4b). A main effect of emotion on N1 latency emerged [*F*(2,150) = 4.38; *p* < 0.05; *η*_p_^2^ = 0.06], but post-hoc t-tests did not indicate significant differences between emotions. However, there was a main effect of group [*F*(2,75) = 7.74; *p* < 0.001; *η*_p_^2^ = 0.17], with post-hoc t-tests indicating significantly shorter N1 latencies in the DPG (110.3 ms; SE 2.0 ms) than in the CCG (118.4 ms; SE 1.9 ms; *p* < 0.01) and the NCG (121.7 ms; SE 2.3 ms; *p* < 0.005), while the CCG and NCG did not differ (*p* = 0.28).

N170. There was a main effect of emotion on N170 amplitude [*F*(2,150) = 9.34; *p* < 0.001; *η*_p_^2^ = 0.11; Fig. 4c], with larger amplitudes to fearful faces (− 1.64 µV; SE 0.52 µV) compared to neutral faces (− 1.00 µV; SE 0.49 µV; *p* < 0.01) and sad faces (− 0.76 µV; 0.44 µV; *p* < 0.001), whereas the N170 amplitudes to the latter two emotions did not differ (*p* = 0.27). No main effect of group and no group × emotion interaction emerged on N170 amplitude (both *F*s < 0.42; both *p*s > 0.66; both *η*_p_^2^ < 0.02). However, there were main effects of emotion [*F*(2,150) = 4.12; *p* < 0.05; *η*_p_^2^ = 0.05] and group [*F*(2,75) = 4.95; *p* < 0.01; *η*_p_^2^ = 0.12] on N170 latency. This was shorter to neutral faces (159.8 ms; SE 1.5 ms) than to fearful faces (161.8 ms; SE 1.4 ms; *p* < 0.01), whereas N170 latency to sad faces (160.9 ms; SE 1.4 ms) did not differ from that to the two other emotions (both *p*s > 0.22). Furthermore, N170 latency was shorter in the DPG (155.1 ms; SE 2.3 ms) than in the CCG (162.0 ms; SE 2.2 ms; *p* < 0.05) and the NCG (165.4 ms; SE 2.6 ms; *p* < 0.05), whereas CCG and NCG did not differ (*p* = 0.31).

P2. There was no main effect of group and no interaction (both *F*s < 0.57; both *p*s > 0.56; both *η*_p_^2^ < 0.02), but a main effect of emotion on P2 mean amplitude emerged [*F*(2,150) = 3.77; *p* < 0.05; *η*_p_^2^ = 0.05; Fig. 4c], with larger amplitudes in fearful faces (12.93 µV; SE 0.59 µV) than in neutral faces (12.31 µV; SE 0.52 µV; *p* < 0.05). P2 amplitudes to sad faces (12.45 µV; SE 0.57 µV) did not differ from fearful or neutral faces (both *p* > 0.12). In addition, a main effect of emotion on P2 latency emerged [*F*(2,150) = 4.69; *p* < 0.05; *η*_p_^2^ = 0.06], with latencies being longer in neutral faces (300.7 ms; SE 3.0 ms) than in fearful faces (295.5 ms; 3.2 ms; *p* < 0.05) and sad faces (295.2 ms; SE 3.2 ms; *p* < 0.05); latencies in fearful and sad faces did not differ (*p* = 0.91). There was no significant main effect of group [*F*(2,75) = 2.54; *p* = 0.09; *η*_p_^2^ = 0.06], and no significant interaction.

Nc. There were no main effects of group, no main effects of emotion, and no group × emotion interactions on Nc mean amplitude or latency (all *F*s < 1.96; all *p*s > 0.14; all *η*_p_^2^ < 0.02; Fig. 4a).

*Additional observation* N170 latency (averaged across all emotional faces) correlated significantly with the SDQ subscale emotional problems (*r* = − 0.34, *p* = 0.002), but not with the other four SDQ subscales (SDQ conduct problems, SDQ hyperactivity, SDQ peer interaction, SDQ prosocial behavior; all *p*s > 0.13). The same applied for the relationship of N1 latency, which also correlated with the SDQ subscale emotional problems (*r* = − 0.31, *p* = 0.006), but not with the other subscales.

## Discussion

In the present study, we tested whether task-irrelevant emotional faces have an effect on attentional performance in children and adolescents with dysregulation profile captured by the SDQ, using a modified ANT paradigm and ERP measures. The ANT results indicate that participants with DP showed a less efficient orienting network compared to the two control groups. As the orienting network is involved in shifting attention to particular locations, less efficiency might therefore allude to DP patients’ impairment in shifting attention to a new location, which in turn may indicate an imbalance between stimulus-driven (exogenous) and goal-directed (endogenous) attentional processes in this network (Meyer et al. 2018). The DP group did not differ from the other groups in any other attentional network, suggesting that DP does not affect attentional functions in general.

The analysis of the ERP data revealed differences regarding the processing of emotional faces: Latencies of both N1 and N170 were significantly shorter in the DP group relative to non-DP controls. Such differences were not found in P1 latency or in P2 and Nc latencies. Moreover, there were no significant group differences in ERP amplitudes. N1 and N170 to facial stimuli have been associated with early processes of selective attention and, more specifically, with the structural decoding of facial expression (e.g., Bentin et al. 1996; Eger et al. 2003; Pizzagalli et al. 1999; Righart and de Gelder 2008). Thus, the shorter N1/N170 latencies in the DP group suggest a faster processing of emotional content compared to non-DP controls at a quite early stage of face processing. From a more individual perspective of emotional skills, it has been proposed that the N170 is sensitive to a person’s emotional sensitivity and expressivity (Meaux et al. 2014). More specifically, a significant negative association was found between N170 latency and the Emotional Sensitivity (ES) subscale score of the Social Skills Inventory (SSI; Riggio 1986), suggesting that persons with shorter N170 latencies show enhanced skills in decoding and understanding emotional messages. On the other side of the coin, however, it has been pointed out that persons with high ES scores are more “concerned with and vigilant in observing the nonverbal emotional cues of others”, and that since they are “able to decode emotional communication rapidly and efficiently, they may be more susceptible to becoming emotionally aroused by others” (Riggio 1986, p. 651; see also Friedman and Riggio 1981). This could also be the case with our DP patients. In contrast to our hypothesis, ERP differences between groups did not depend on the emotional expression of the faces at all. In particular, the observed differences in N1/N170 latencies were equally pronounced for negative and neutral emotions, suggesting that the faster face processing of DP patients was evidently not modulated by the emotional content per se. This observation is in line with the behavioral ANT findings, according to which group differences also did not depend on the emotional content of the faces. Given that only negative (fearful and sad) and neutral faces were used as pre-stimuli in the present study, the question remains open whether the observed differences in N1/N170 latencies between groups are confined to negative emotions or can also be generalized to positive emotions. Regarding the N1 results, it would be important to test in future studies whether shorter latencies are also observed to non-facial (emotional) stimuli. However, the fact that latency differences were only found for N1/N170, and not for the earlier P1 and the later P2 and Nc components, might indicate that specific processes of facial encoding are affected in DP patients.

As a mere side effect, we found clear-cut effects of emotion on N170 and P2, both of which were larger in amplitude to fearful faces than to neutral faces. The modulation of the N170 amplitude is in line with other studies using emotional faces as distractors and showing robust emotion effects at the level of the N170 component (Hinojosa et al. 2015). We could replicate results on the N170 for fearful facial expressions used in our study (Almeida et al. 2014; Baggott et al. 2011; Denefrio et al. 2017; Jiang et al. 2009; MacNamara et al. 2012; Müller-Bardorff et al. 2016; Peschard et al. 2013). Emotional effects seem to have marked influence on the P2, which was also shown here (e.g., Calvo et al. 2013; Carretié et al. 2012; Jaworska et al. 2012). In addition, latency effects emerged, with longer N170 latencies and shorter P2 latencies to fearful than to neutral faces. These effects are in line with a number of previous findings (e.g., Batty and Taylor 2003; Eimer and Holmes 2007; Leppänen et al. 2007; Righart and de Gelder 2008; Meaux et al. 2014), and demonstrate the impact of emotional content on the structural encoding of faces as well as visual attention to emotional stimuli.

Support for our results comes from studies in patients reporting severe mood dysregulation (SMD). There is a great overlap of symptoms between patients with the DP described here and patients with SMD, e.g., regarding irritability and mood swings as well as depressive symptoms (Holtmann et al. 2011; Juksch et al. 2011). fMRI research in SMD patients showed a hypersensitivity towards negative facial stimuli (Tseng et al. 2016). The authors linked these findings to the patients’ higher irritability and anxiety symptoms. A further study by Hommer et al. (2014) showed that children and adolescents with SMD have an attention bias towards threatening faces, which also corresponds to our findings. Future research might therefore address attentional bias modification as a potential treatment for children and adolescents with DP.

When interpreting the present results, a few limiting factors have to be considered: The study was conducted in a naturalist inpatient treatment setting and is observational in nature. We did not match controls and patients according to age, sex and IQ. Therefore, differences in the samples emerged regarding gender distribution and IQ, potentially limiting the comparability of the results. Furthermore, the healthy controls were only screened for mental disorders, and did not undergo a structured clinical assessment; accordingly, they may have had mental health problems which we did not capture with the screening, thus potentially impacting the results. Finally, our findings show that N170 and N1 latencies correlate with the SDQ subscale “emotional problems”, suggesting that emotional problems have a greater influence on our data than do conduct problems or hyperactivity. Further studies are needed to further explore the reciprocal relationship of emotion and attention in these patient groups.

In conclusion, the present study aimed to link attentional processes and emotion regulation in patients with DP, contrasting behavioral and EEG data of a clinical and healthy control group in a modified version of the ANT. Two characteristic traits of adolescents with DP emerged: While executive attentional performance was, contrary to expectations, only slightly behaviorally modulated by emotional distractors, adolescents with DP showed a severe deficient stimulus-driven orienting function. In addition the ERPs provided evidence for a faster processing of emotional distractors, which, however, is accompanied by a lower processing level.

## References

[CR1] Achenbach TM (1991). Manual for Child Behavior Checklist/ 4–18 and 1991 Profile.

[CR2] Aebi M, Metzke CW, Steinhausen H-C (2019). Predictors and outcomes of self-reported dysregulation profiles in youth from age 11 to 21 years. Eur Child Adolesc Psychiatry.

[CR3] Almeida PR, Ferreira-Santos F, Vieira JB, Moreira PS, Barbosa F, Marques-Teixeira J (2014). Dissociable effects of psychopathic traits on cortical and subcortical visual pathways during facial emotion processing: an ERP study on the N170. Psychophysiology.

[CR4] Althoff RR, Ayer LA, Rettew DC, Hudziak JJ (2010). Assessment of dysregulated children using the Child Behavior Checklist: a receiver operating characteristic curve analysis. Psychol Assess.

[CR5] Ayer L, Althoff R, Ivanova M, Rettew D, Waxler E, Sulman J, Hudziak J (2009). Child Behavior Checklist Juvenile Bipolar Disorder (CBCL-JBD) and CBCL Posttraumatic Stress Problems (CBCL-PTSP) scales are measures of a single dysregulatory syndrome. J Child Psychol Psychiatry.

[CR6] Baggott S, Palermo R, Fox AM (2011). Processing emotional category congruency between emotional facial expressions and emotional words. Cogn Emot.

[CR7] Batty M, Taylor MJ (2003). Early processing of the six basic facial emotional expressions. Cogn Brain Res.

[CR8] Becker A, Wang B, Kunze B, Otto C, Schlack R, Hölling H (2018). Normative data of the self-report version of the German Strengths and Difficulties Questionnaire in an epidemiological setting. Z Kinder Jugendpsychiatr Psychother.

[CR9] Bentin S, Allison T, Puce A, Perez E, McCarthy G (1996). Electrophysiological studies of face perception in humans. J Cogn Neurosci.

[CR10] Biederman J, Petty CR, Monuteaux MC, Evans M, Parcell T, Faraone SV, Wozniak J (2009). The child behavior checklist-pediatric bipolar disorder profile predicts a subsequent diagnosis of bipolar disorder and associated impairments in ADHD youth growing up. A longitudinal analysis. J Clin Psychiatry.

[CR11] Calvo MG, Marrero H, Beltrán D (2013). When does the brain distinguish between genuine and ambiguous smiles? An ERP study. Brain Cogn.

[CR12] Carballo JJ, Serrano-Drozdowskyj E, García Nieto R, Díaz de Neira-Hernando M, Pérez-Fominaya M (2014). Prevalence and correlates of psychopathology in children and adolescents evaluated with the strengths and difficulties questionnaire dysregulation profile in a clinical setting. Psychopathology.

[CR13] Carretié L (2014). Exogenous (automatic) attention to emotional stimuli: a review. Cogn Affect Behav Neurosci.

[CR14] Carretié L, Mercado F, Tapia M, Hinojosa JA (2001). Emotion, attention, and the “negativity bias”, studied through event-related potentials. Int J Psychophysiol.

[CR15] Carretié L, Kessel D, Carboni A, López-Martín S, Albert J, Tapia M (2012). Exogenous attention to facial *vs* non-facial emotional visual stimuli. Soc Cogn Affective Neurosci.

[CR16] Cohen N, Henik A, Mor N (2011). Can emotion modulate attention? Evidence for reciprocal links in the attentional network test. Exp Psychol.

[CR17] Denefrio S, Simmons A, Jha A, Dennis-Tiwary TA (2017). Emotional cue validity effects: the role of neurocognitive responses to emotion. PLoS ONE.

[CR18] Dennis TA, Chen C-C (2007). Emotional face processing and attention performance in three domains. Neurophysiological mechanisms and moderating effects of trait anxiety. Int J Psychophysiol: Off J Int Org Psychophysiol.

[CR19] Dennis TA, Chen C-C, McCandliss BD (2008). Threat-related attentional biases: an analysis of three attention systems. Depression Anxiety.

[CR20] Dennis TA, Malone MM, Chen C-C (2009). Emotional face processing and emotion regulation in children: An ERP study. Dev Neuropsychol.

[CR21] Deutz MHF, Shi Q, Vossen HGM, Huijding J, Prinzie P, Deković M (2018). Evaluation of the strengths and difficulties questionnaire-dysregulation profile (SDQ-DP). Psychol Assess.

[CR22] Deutz MHF, Geeraerts SB, Belsky J, Deković M, van Baar AL, Prinzie P, Patalay P (2020). General psychopathology and dysregulation profile in a longitudinal community sample: stability, antecedents and outcomes. Child Psychiatry Hum Dev.

[CR23] Eger E, Jedynak A, Iwaki T, Skrandies W (2003). Rapid extraction of emotional expression: evidence from evoked potential fields during brief presentation of face stimuli. Neuropsychologia.

[CR24] Eimer M, Holmes A (2002). An ERP study on the time course of emotional face processing. NeuroReport.

[CR25] Eimer M, Holmes A (2007). Event-related brain potential correlates of emotional face processing. Neuropsychologia.

[CR26] Eriksen BA, Eriksen CW (1974). Effects of noise letters upon the identification of a target letter in a nonsearch task. Percept Psychophys.

[CR27] Fan J, McCandliss BD, Sommer T, Raz A, Posner MI (2002). Testing the efficiency and independence of attentional networks. J Cogn Neurosci.

[CR28] Friedman HS, Riggio RE (1981). Effect of individual differences in nonverbal expressiveness on transmission of emotion. J Nonverbal Behav.

[CR29] Goodman R, Meltzer H, Bailey V (1998). The strengths and difficulties questionnaire. A pilot study on the validity of the self-report version. Eur Child Adolescent Psychiatry.

[CR30] Heeren A, Mogoaşe C, McNally RJ, Schmitz A, Philippot P (2015). Does attention bias modification improve attentional control? A double-blind randomized experiment with individuals with social anxiety disorder. J Anxiety Disord.

[CR31] Hinojosa JA, Mercado F, Carretié L (2015). N170 sensitivity to facial expression: a meta-analysis. Neurosci Biobehav Rev.

[CR32] Holtmann M, Bölte S, Goth K, Döpfner M, Plück J, Huss M (2007). Prevalence of the child behavior checklist-pediatric bipolar disorder phenotype in a German general population sample. Bipolar Disord.

[CR33] Holtmann M, Goth K, Wöckel L, Poustka F, Bölte S (2008). CBCL-pediatric bipolar disorder phenotype Severe ADHD or bipolar disorder?. J Neural Transmission.

[CR34] Holtmann M, Buchmann AF, Esser G, Schmidt MH, Banaschewski T, Laucht M (2010). The child behavior checklist-dysregulation profile predicts substance use, suicidality, and functional impairment. A longitudinal analysis. J Child Psychol Psychiatry, Allied Disciplines.

[CR35] Holtmann M, Duketis E, Goth K, Poustka L, Boelte S (2010). Severe affective and behavioral dysregulation in youth is associated with increased serum TSH. J Affect Disord.

[CR36] Holtmann M, Becker A, Banaschewski T, Rothenberger A, Roessner V (2011). Psychometric validity of the strengths and difficulties questionnaire-dysregulation profile. Psychopathology.

[CR37] Hommer RE, Meyer A, Stoddard J, Connolly ME, Mogg K, Bradley BP (2014). Attention bias to threat faces in severe mood dysregulation. Depress Anxiety.

[CR38] Hudziak JJ, Althoff RR, Derks EM, Faraone SV, Boomsma DI (2005). Prevalence and genetic architecture of child behavior checklist-juvenile bipolar disorder. Biol Psychiat.

[CR39] Jasper HH (1924) The ten twenty electrode system of the international federation. Report of the Committee on the Methods of Clinical Examination in Electroencephalography 370–375

[CR40] Jaworska N, Blier P, Fusee W, Knott V (2012). The temporal electrocortical profile of emotive facial processing in depressed males and females and healthy controls. J Affect Disord.

[CR41] Jiang Y, Shannon RW, Vizueta N, Bernat EM, Patrick CJ, He S (2009). Dynamics of processing invisible faces in the brain: Automatic neural encoding of facial expression information. Neuroimage.

[CR42] Jucksch V, Salbach-Andrae H, Lenz K, Goth K, Döpfner M, Poustka F (2011). Severe affective and behavioural dysregulation is associated with significant psychosocial adversity and impairment. J Child Psychol Psychiatry.

[CR43] Legenbauer T, Hübner J, Pinnow M, Ball A, Pniewski B, Holtmann M (2018). Proper emotion recognition, dysfunctional emotion regulation. The mystery of affective dysregulation in adolescent psychiatric inpatients. Zeitschrift Kinder- Jugendpsychiatrie Psychotherapie.

[CR44] Leppänen JM, Moulson MC, Vogel-Farley VK, Nelson CA (2007). An ERP study of emotional face processing in the adult and infant brain. Child Dev.

[CR45] Lijffijt M, Lane SD, Meier SL, Boutros NN, Burroughs S, Steinberg JL (2009). P50, N100, and P200 sensory gating. Relationships with behavioral inhibition, attention, and working memory. Psychophysiology.

[CR46] MacNamara A, Schmidt J, Zelinsky GJ, Hajcak G (2012). Electrocortical and ocular indices of attention to fearful and neutral faces presented under high and low working memory load. Biol Psychol.

[CR47] Mangun GR (1995). Neural mechanisms of visual selective attention. Psychophysiology.

[CR48] Meaux E, Roux S, Batty M (2014). Early visual ERPs are influenced by individual emotional skills. Soc Cogn Affective Neurosci.

[CR49] Meyer KN, Du F, Parks E, Hopfinger JB (2018). Exogenous vs. endogenous attention: shifting the balance of fronto-parietal activity. Neuropsychologia.

[CR50] Morawetz C, Baudewig J, Treue S, Dechent P (2010). Diverting attention suppresses human amygdala responses to faces. Front Hum Neurosci.

[CR51] Müller-Bardorff M, Schulz C, Peterburs J, Bruchmann M, Mothes-Lasch M, Milner W, Straube T (2016). Effects of emotional intensity under perceptual load: an event-related potentials (ERPs) study. Biol Psychol.

[CR52] Nelson CA, de Haan M (1996). Neural correlates of infants’ visual responsiveness to facial expressions of emotion. Dev Psychobiol.

[CR53] Peschard V, Philippot P, Joassin F, Rossignol M (2013). The impact of the stimulus features and task instructions on facial processing in social anxiety: an ERP investigation. Biol Psychol.

[CR54] Pizzagalli D, Regard M, Lehmann D (1999). Rapid emotional face processing in the human right and left brain hemispheres. An ERP study. NeuroReport Rapid Commun Neurosci Res.

[CR55] Pourtois G, Schettino A, Vuilleumier P (2013). Brain mechanisms for emotional influences on perception and attention. What is magic and what is not. Biol Psychol.

[CR56] Rich BA, Schmajuk M, Perez-Edgar KE, Fox NA, Pine DS, Leibenluft E (2007). Different psychophysiological and behavioral responses elicited by frustration in pediatric bipolar disorder and severe mood dysregulation. Am J Psychiatry.

[CR57] Richards JE, Reynolds GD, Courage ML (2010). The neural bases of infant attention. Curr Dir Psychol Sci.

[CR58] Riggio RE (1986). Assessment of basic social skills. J Pers Soc Psychol.

[CR59] Righart R, de Gelder B (2008). Rapid influence of emotional scenes on encoding of facial expressions. An ERP study. Soc Cogn Affective Neurosci.

[CR60] Rueda MR, Fan J, McCandliss BD, Halparin JD, Gruber DB, Lercari LP, Posner MI (2004). Development of attentional networks in childhood. Neuropsychologia.

[CR61] Tottenham N, Tanaka JW, Leon AC, McCarry T, Nurse M, Hare TA (2009). The NimStim set of facial expressions. Judgments from untrained research participants. Psychiatry Res.

[CR62] Tseng W-L, Thomas LA, Harkins E, Pine DS, Leibenluft E, Brotman MA (2016). Neural correlates of masked and unmasked face emotion processing in youth with severe mood dysregulation. Soc Cogn Affective Neurosci.

[CR63] Wang B, Brueni LG, Isensee C, Meyer T, Bock N, Ravens-Sieberer U (2018). Predictive value of dysregulation profile trajectories in childhood for symptoms of ADHD, anxiety and depression in late adolescence. Eur J Child Adolescent Psychiatry.

[CR64] Wang B, Eastwood PR, Becker A, Isensee C, Wong JW, Huang YR-C (2019). Concurrent developmental course of sleep problems and emotional/behavioral problems in childhood and adolescence as reflected by the dysregulation profile. Sleep.

[CR65] Yamaguchi S, Onada K (2012). Interaction between emotion and attention systems. Front Neurosci.

